# Using Autoregressive Integrated Moving Average (ARIMA) Modelling to Forecast Symptom Complexity in an Ambulatory Oncology Clinic: Harnessing Predictive Analytics and Patient-Reported Outcomes

**DOI:** 10.3390/ijerph18168365

**Published:** 2021-08-07

**Authors:** Linda Watson, Siwei Qi, Andrea DeIure, Claire Link, Lindsi Chmielewski, April Hildebrand, Krista Rawson, Dean Ruether

**Affiliations:** 1 Applied Research and Patient Experience, Cancer Research and Analytics, Cancer Care Alberta—Alberta Health Services, Calgary, AB T2S 3C3, Canada; Siwei.Qi@albertahealthservices.ca (S.Q.); Andrea.Deiure@albertahealthservices.ca (A.D.); Claire.Link@albertahealthservices.ca (C.L.); Lindsi.Chmielewski@albertahealthservices.ca (L.C.); April.Hildebrand@albertahealthservices.ca (A.H.); 2 Quality, Safety & Practice Integration, Cancer Care Alberta—Alberta Health Services, Calgary, AB T2S 3C3, Canada; Krista.Rawson@albertahealthservices.ca; 3 Medical Oncology, Cancer Care Alberta—Alberta Health Services, Calgary, AB T2S 3C3, Canada; Dean.Ruether@albertahealthservices.ca; 4 Department of Oncology, Cumming School of Medicine, Foothills Campus, University of Calgary, Calgary, AB T2N 4N1, Canada

**Keywords:** patient-reported outcomes, symptom complexity levels, forecasting model, predictive analytics, ARIMA, clinic scheduling, staff allocation

## Abstract

An increasing incidence of cancer has led to high patient volumes and time challenges in ambulatory oncology clinics. By knowing how many patients are experiencing complex care needs in advance, clinic scheduling and staff allocation adjustments could be made to provide patients with longer or shorter timeslots to address symptom complexity. In this study, we used predictive analytics to forecast the percentage of patients with high symptom complexity in one clinic population in a given time period. Autoregressive integrated moving average (ARIMA) modelling was utilized with patient-reported outcome (PRO) data and patient demographic information collected over 24 weeks. Eight additional weeks of symptom complexity data were collected and compared to assess the accuracy of the forecasting model. The predicted symptom complexity levels were compared with observation data and a mean absolute predicting error of 5.9% was determined, indicating the model’s satisfactory accuracy for forecasting symptom complexity levels among patients in this clinic population. By using a larger sample and additional predictors, this model could be applied to other clinics to allow for tailored scheduling and staff allocation based on symptom complexity forecasting and inform system level models of care to improve outcomes and provide higher quality patient care.

## 1. Introduction

The rapid expansion of electronic health records (EHR) and an increase in the collection of electronic clinical data and analytics in health care settings [1] has resulted in opportunities to improve efficiencies, reduce cost, and guide system transformation [2]. By using historical data and statistical modelling, it is now possible to make robust predictions about how to improve patient outcomes and provide a higher standard of care [3]. Data-rich health care environments such as oncology settings are a natural fit for predictive analytics, but the use of such analytic strategies is sparse in this area, especially when forecasting prevalence of symptoms [4]. 

Effective symptom management is a daunting but important challenge in ambulatory oncology settings [5,6,7]. As a standard component of care, the collection of longitudinal patient reported outcomes (PROs) is pivotal for identifying patient needs and improving outcomes such as symptom control, health-related quality of life, and overall functioning [8,9,10,11]. Timely and consistent review of patient concerns and symptoms by clinicians, in outpatient settings where demands for time are ever increasing, remains to be a challenge [6]. A recent review of the literature regarding the use of PROs in routine cancer care found that the most common barrier cited by health care providers was insufficient time to interpret, action on, and discuss PRO data with patients during clinics, and the most common service-level barrier was a lack of integration of PROs into clinical workflows [12]. 

Cancer Care Alberta (CCA) is the provincial ambulatory oncology program in Alberta, Canada. Alberta is home to 4.4 million people [13] with an expected annual cancer incidence of 23,424 by 2023 [14]. CCA provides publicly funded, comprehensive cancer care services through a network of 17 ambulatory facilities. Routine collection of PRO data is integrated into clinical workflows to assist clinicians in identifying and supporting targeted, timely symptom management [15]. The provincial PRO questionnaire, named Putting Patients First (PPF), includes two standardized measures: the Revised Edmonton Symptom Assessment System (ESAS-r) and the Canadian Problem Checklist (CPC) [16]. Currently, patients fill out the paper PPF in the waiting room and it is reviewed during their clinic visit. Then, the PRO data and associated clinical responses are entered into the electronic medical record (EMR) after the clinical encounter, allowing for digital PROs reporting and analytics. As CCA does not yet have a patient portal which, once available, will allow direct entry of PRO data by patients prior to the clinical encounter, clinic staff only have access to historical digital PRO data at this time.

### 1.1. Symptom Complexity Algorithm

**Figure 1:**
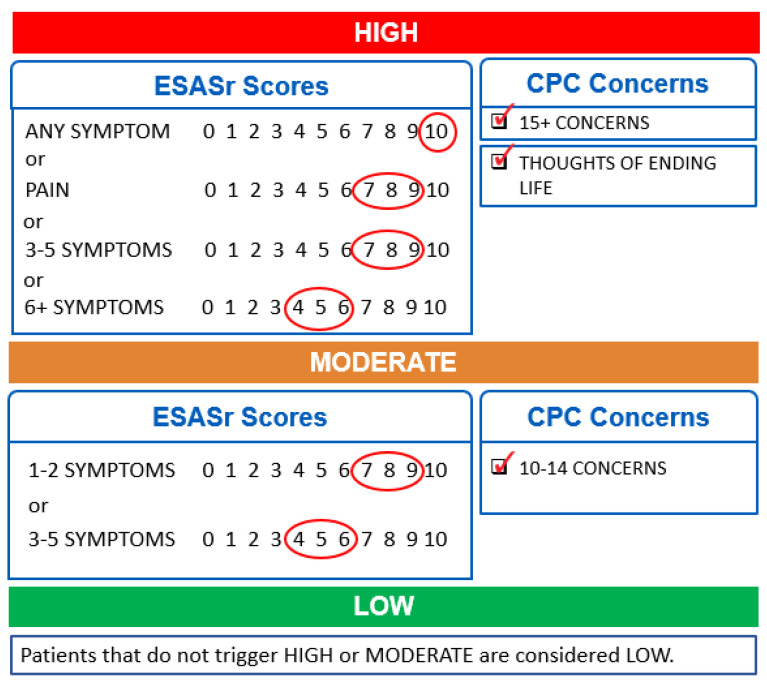
Symptom complexity algorithm.

Clinicians have reported that patients with higher symptom burden generally need more time in a clinic [17,18,19], which aligns with published studies on obstacles to using PROs in routine practice, suggesting there are broad issues with interpretation and time limitations for the utilization of PRO data in clinical care encounters [20]. To support the capacity of clinicians to pragmatically utilize PRO data in practice, CCA developed a symptom complexity algorithm capable of generating a visual complexity flag for the patient’s encounter. The algorithm considers both the severity of symptoms and the number of concerns reported on the patient’s last completed PPF and assigns a symptom complexity score (low/green, moderate/yellow or high/red) for the encounter if any one of the criteria is met (criteria for each score are shown in Figure 1). This clinician-facing summary score was designed to support clinical teams in their efforts to tailor their time allocation and approach to each patient. The algorithm has shown satisfactory accuracy, good discrimination, and calibration in classifying symptom complexity levels among cancer patients [21].

### 1.2. Scheduling Clinic Time and Allocating Staff

The increasing incidence of cancer in Alberta [14], along with a trend of treating patients in ambulatory clinics for a longer period of time over the course of their disease [22], has made it challenging to manage the high volume of patients and address all their symptoms and concerns within the allotted clinic schedule. Clinic space and time is fixed, making it essential to optimize the time within each patient encounter. Cancer patients with high symptom complexity may benefit from an increased level of care in the clinic [17,18,19], which is a challenge when there is no capacity to tailor the length of a clinic visit to reflect a patient’s increased needs. By combining the symptom complexity algorithm with predictive analytics, a novel opportunity exists to predict the distribution of symptom complexity among patients in a particular clinical cohort and, accordingly, plan for the appropriate scheduling of patient appointments in the future. 

### 1.3. Purpose of the Study 

The purpose of this study was to test if predictive analytics could be used to accurately forecast the percentage of patients within an ambulatory oncology clinic population who would present with high symptom complexity in a given time period. This was done by using retrospective PRO data from one cancer clinic in CCA and autoregressive integrated moving average (ARIMA) modelling. This type of forecasting, if used with a larger sample and extended time period, has the potential to enable proactive appointment scheduling, by allowing the appropriate number of longer visits to be booked into a clinic [23], giving the clinician more time to address patients with high levels of symptom complexity. Similarly, there is potential to forecast the number of appointments which could be shortened, deferred, or converted to virtual encounters for patients with low symptom complexity [12]. This initial study, however, only aimed to test the feasibility of this type of predictive model to determine if further investigation and expansion of the model was worth pursuing. 

## 2. Materials and Methods

### 2.1. Study Setting and Design

This retrospective study was carried out in a weekly treatment clinic located at a tertiary cancer centre in Alberta, Canada. The clinical cohort of patients were predominantly neuroendocrine/endocrine (NET/END) and genitourinary (GU) cancer patients. Most patients in this clinic had metastatic disease and were over 60 years old. Consistent clinic staff included a medical oncologist, a registered nurse, and a general practitioner, with access to other staff occasionally. In the study timeframe, approximately 73.5% of patients in this clinic had filled out a PPF, consistent with the provincial average (75%). 

For this study, only patients who visited the clinic and completed a PPF were included. Individual weeks were used as the unit of time because the clinic occurred once a week. Historical PRO data and demographics were collected between 7 August 2019 and 29 January 2020. Eight additional weeks of data were collected between 5 February 2020 and 25 March 2020 to assess the accuracy of the forecasting model. In total, 32 weeks of historical PRO data were considered for this study. The selection of potential predictors and timeframe of the data was motivated by what was accessible within a larger health system utilization dataset that was readily available to the research team. 

### 2.2. Ethics Approval 

This initiative did not involve any patient contact. The Alberta Innovates ARECCI Screening Tool and Ethics Guideline Tool [24] were used to determine that the study qualified as quality improvement with minimal risk, and full ethics approval was not required. 

### 2.3. Data Analysis

Patient characteristics (age, sex, and tumour group) were categorized at the clinic level by visit date. The tumour group was categorized as NET/END, GU, and other. A symptom complexity level was assigned for each encounter based on PRO data and the symptom complexity algorithm. As we did not have a full year of data, a seasonal ARIMA model (SARIMA) was not possible, and therefore the seasonal variable was constructed categorically as three levels, i.e., spring/summer, fall, and winter, to fit our observation window. This variable was included based on the existing literature demonstrating that cancer patients’ symptoms, including depression, anxiety, well-being, and appetite, are linked to seasonality [25,26].

Time series analysis forecasting the percentage of patients who would have high symptom complexity was carried out using ARIMA modelling, an established procedure. ARIMA modelling has progressively gained popularity in cancer research, mainly to forecast cancer incidence [27,28,29,30]. Our study aimed to expand the use of this method in the field of cancer symptom prediction. To the best of our knowledge, this is the first study of this type in this field. 

### 2.4. Data Structure and Model Fit

We determined that the data used in this study were in a stationary format. Using season, age, sex, and tumour group as predictors, we evaluated a number of commonly used ARIMA models with different structures [27,28,29,31,32]. Table 1 lists the goodness-of-fit measures used to assess and compare the different models. We found that ARIMA (0, 0, 1) had the smallest normalized Bayesian information criteria (BIC) and a non-significant Ljung-box statistic, indicating that this structure fit the data better than other structures. Model performance on the historical 24 weeks of data used to fit the model was summarized by mean absolute error (MAE) and root squared mean error (RSME). The selected ARIMA model (0, 0, 1) had the lowest MAE and RMSE. 

**Figure 2:**
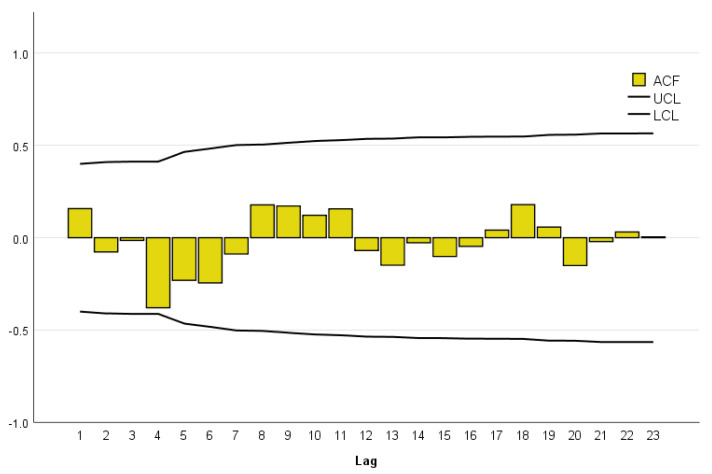
Residual ACF of the ARIMA model (0, 0, 1).

Following model selection, the autocorrelation function (ACF) was performed to conduct model diagnostic checking. We plotted the ACF (Figure 2), which indicated a reasonable model fit. These tests, together, verified that our chosen AR, MA, and differencing (I) were appropriate. 

### 2.5. Forecasting and Forecast Accuracy

Forecasting accuracy was assessed using mean absolute predicting error (MAPE) by comparing the forecasted values with 8 weeks of actual clinic visit data (spanning from 5 February 2020 to 25 March 2020). We used a threshold of 5% as acceptable for MAE and MAPE based on how the errors impacted application of the model, meaning that if the model was applied in a clinic, they would be able to reasonably manage 5% over- or under- predicting. The data were exported into SPSS Statistics, version 25 (SPSS Statistics for Windows, Version 25.0. IBM Corp: Armonk, NY, USA) for analysis, with statistical significance set a priori at *p* < 0.05.

## 3. Results

### 3.1. Sample Characteristics

Between August 2019 and January 2020, there was a total number of 524 visits to the clinic, with 385 PPFs collected from these visits. Table 2 presents the mean age, distribution of sex and tumour groups, and percentage of symptom complexity levels (low, moderate, and high) for these patients, as calculated from the PRO data for each week. All variables were aggregated to the clinic level for the purpose of analysis.

Most patients seen in this clinic were male, making up 70% to 100% of patients across the observation period. The average age of patients ranged from 61.8 to 73.2 years. The GU tumour group had the largest share of patients (from 35.3% to 83.3%). The percentage of patients with high symptom complexity level varied per week, ranging from a low of 0% on 27 November 2019 to a high of 30% on 4 September 2019. The percentage of patients with low symptom complexity level ranged from 45.0% to 84.2%.

### 3.2. Forecasting and Forecast Accuracy

**Figure 3:**
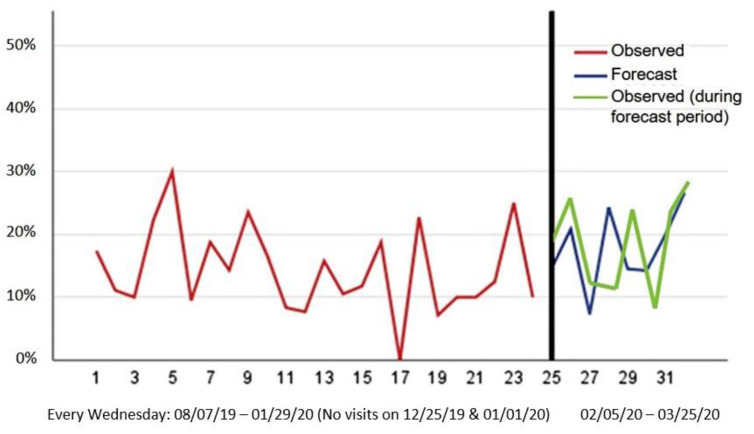
Forecasting percentage of high symptom complexity.

Using the selected ARIMA model (0, 0, 1), we obtained forecasts of the percentage of patients with high symptom complexity levels for February 2020 and March 2020, based on the data from August 2019 to January 2020. The results are presented in Figure 3, with the weekly observed percentage of high symptom complexity levels indicated by the red line, the forecasted weekly percentage of high symptom complexity levels indicated by the blue line, and the observed weekly percentage during the forecasting period indicated by the green line. The forecasted percentage of high symptom complexity levels ranged from 7.3% to 26.7%. 

Finally, to evaluate the forecasting accuracy, we calculated the absolute prediction error by comparing the forecasted percentages to the actual data collected from the forecasted period (see Figure 3); the results are presented in Table 3. The MAPE was 5.9%, which is just above our acceptable threshold of 5%. The largest discrepancy between the forecasts and the actual observations occurred on 26 February 2020, when the model predicted that an additional 11.8% of patients would have high symptom complexity. The smallest discrepancy occurred on 25 March 2020, when the model predicted only 1.9% fewer patients would have high symptom complexity. 

## 4. Discussion

As the collection of electronic PROs and other health information increases in clinical settings [1], the use of statistical modelling and predictive analytics can provide a unique way of forecasting the prevalence of symptoms and concerns in patient populations [3]. In cancer care, this has created an opportunity to understand how symptom complexity levels could inform clinical processes, leading to improved symptom management, patient outcomes and ultimately a higher standard of care [3]. 

This study sought to use predictive analytics with retrospective PRO data and ARIMA modelling to forecast the percentage of patients within an ambulatory clinic population who would present with high symptom complexity in a specific time period. The forecasting model that was developed and tested produced results with a satisfactory level of accuracy, both in the fit to the data (MAE = 4.0%) and in the forecasting of future data (MAPE = 5.9%), confirming the model’s potential for establishing a weekly prediction of symptom complexity in this patient cohort. Given the relatively short timeframe, these findings are encouraging, as our MAE was below the 5% threshold, and our MAPE was only slightly above. We anticipate that expanding the model by including more data over a longer period of time (one year at minimum), enabling the inclusion of a true seasonal element within the model, would help to reduce the error. 

It is important to recognize that the error varied week to week, with larger discrepancies between actual and predicted data during the weeks of 26 February 2020 and 4 March 2020. This could be due to the outbreak of COVID-19 in Alberta, which occurred in February 2020 and caused considerable disruptions within cancer care [33,34]. Healthier patients, including those with low symptom complexity, may have been shifted to a virtual appointment and it has been shown that few virtual patients completed the PPF [34]. Therefore, these patients would not have been included in the dataset, resulting in fewer low complexity patients being represented. Furthermore, the added stressors of the pandemic may have resulted in some level of response bias regarding the PPF, for example, some patients may have reported uncharacteristically high levels of anxiety and depression [35]. Some of these patients may have been outliers, if they had highly heightened symptom burdens due to the pandemic. An option is to use the ARIMA model to detect the different types of outliers (such as additive or level shift) and let the model handle them appropriately, possibly by excluding them altogether. This may help lower the MAE, and when we expand the model in the future, using a larger dataset, this option should be utilized to improve accuracy. 

It should be noted that this model could also be used to forecast low and moderate symptom complexity, which was not done in the current study. This would be important to consider when implementing an expanded model for use in clinics in the future. Even with the expanded model, there would inevitably be errors. However, the potential benefits of this type of modelling outweigh the issues presented by the errors. Additionally, recognizing and understanding that errors will occur enables clinical teams to respond appropriately, for example, by always allowing for some unscheduled time within the clinic scheduling template so that patients who require more care than predicted could be managed without clinic overtime. 

### 4.1. Forecasting Symptom Complexity to Improve Clinical Processes

Due to the high variability of patients’ symptoms and needs, equally allotted clinic timeslots for each patient often fail to account for the time needed for in-depth assessments, which is why a symptom-triaged distribution of clinic time is desirable [32]. Fluctuation in time and care demands are constant issues in cancer care, and the quality of scheduling depends on the reliability of staff workload estimation [36]. Cancer patients have unique concerns, needs, and preferences [37,38,39], and should be distinguished, accordingly; this is fundamental for planning efficient and effective clinics [19]. By using ARIMA modelling to forecast the percentage of patients with high symptom complexity on a given clinic date, specific cancer clinics could use prearranged and appropriate time/staffing allocation measures to be better equipped to meet diverse patient needs. Staffing needs in a clinic where many patients are experiencing a high level of symptom complexity differ considerably from a clinic where there are very few patients with high symptom complexity. Advance knowledge would enable the clinic to prepare more effectively, by scheduling a mix of longer and shorter timeslots to match the complexity burden on a specified clinic date. Forecasting the proportion of low complexity patients that require shorter time slots could support more effective use of the total allotted time, while still providing the care needed by all patients seen in the clinic. This is in keeping with person-centred care, i.e., ensuring that the individual needs and concerns of patients are taken into consideration to provide them with the care they need—whether more or less [37,38,39,40]. 

### 4.2. Forecasting Symptom Complexity to Influence Innovative System Level Models of Care

This forecasting model (if expanded) could be proactively utilized in strategic planning to critically evaluate models of care for ambulatory cancer clinics within CCA and other cancer care systems, to optimize practices, and to enhance efficiency while maintaining high quality patient care. More appropriate allocation of time within clinics to provide care tailored to an individual’s needs would benefit patients and the cancer program and improve the overall level of care. This model could be expanded to triage patients to either virtual or in-person appointments based on their individual symptom complexity and care needs. Using the model in this way supports a learning health system (LHS) [41], as CCA is using its own data to adapt workflows to patient needs, ultimately improving practices and outcomes. 

### 4.3. Limitations

This study is not without limitations. We utilized less than a full year of retrospective data (24 weeks instead of 52 weeks), resulting in the inability to model the periodically repeated trend. As our next step, we aim to include the full seasonal element using a time series model such as SARIMA, upon the availability of a larger dataset. Additionally, the model only included patients with a completed PPF, as this was required to determine the patient’s symptom complexity level. This meant that a quarter of patients who visited the clinic in the given time period were excluded. Therefore, the predicted percentage of patients with high symptom complexity could be somewhat higher or lower than the actual clinic complexity, as the model is unable to account for patients with missing PRO data. Our sample size of 385 PPFs is quite small, limiting the accuracy and usability of the model. However, as we only intended to test this initial form of the model, rather than use this model in its current form in a clinic setting, these sample limitations are acceptable. 

The data collected came from one ambulatory cancer clinic, providing care to a specific set of cancer patients with GU and NET/END malignancies, and the model included few predictors. This ARIMA modelling strategy would need to be adjusted in order to be applied to other clinical populations. Additional analyses should be conducted by collecting data from multiple clinics in a variety of settings for a longer period of time. Future research should also consider time-in-motion analysis and the collection of qualitative patient and clinician perspectives to understand how forecasting models could be further optimized and used in cancer clinics to improve patient and care provider experiences alongside clinical efficiencies and processes.

## 5. Conclusions

This study has demonstrated that ARIMA modelling has the potential to forecast patients’ symptom complexity levels at the clinic level. The model showed satisfactory accuracy for an initial test, however, with further modifications, such as including the seasonal element using SARIMA (upon the availability of a larger sample and longer time period), the forecasting accuracy could be improved. An improved and expanded model could potentially be used at organizational levels to inform new and innovative models of care and ultimately provide patients with higher quality care, leading to improved outcomes. 

## Figures and Tables

**Table 1:** Model Comparisons.

Model	Normalized BIC	Ljung-Box Q (18)	Ljung-Box *p*	MAE	RMSE
ARIMA (0, 0, 1)	−4.72	19.66	0.292	0.040	0.056
ARIMA (0, 1, 0)	−3.57	26.65	0.086	0.067	0.104
ARIMA (1, 1, 0)	−3.82	12.66	0.759	0.057	0.089
ARIMA (0, 1, 1)	−3.97	19.98	0.275	0.052	0.080
ARIMA (1, 1, 1)	−3.92	17.58	0.349	0.050	0.076

**Table 2:** Patient characteristics and distribution of symptom complexity at the clinic level.

Clinic Visit Date	Age (M)	Sex (% Male)	TG * (% NET/END)	TG * (%GU)	% Low	% Moderate	% High
07-Aug-2019	66.6	82.6	17.4	56.5	69.6	13.0	17.4
14-Aug-2019	69.6	88.9	22.2	44.4	55.6	33.3	11.1
21-Aug-2019	63.7	70.0	10.0	70.0	70.0	20.0	10.0
28-Aug-2019	61.9	100.0	0.0	77.8	55.6	22.2	22.2
04-Sep-2019	63.7	72.7	13.6	50.0	45.0	25.0	30.0
11-Sep-2019	69.0	71.4	19.0	52.4	66.7	23.8	9.5
18-Sep-2019	66.7	81.3	0.0	81.3	56.3	25.0	18.8
25-Sep-2019	67.9	92.8	14.3	35.7	57.1	28.6	14.3
02-Oct-2019	61.8	64.7	23.5	35.3	47.1	29.4	23.5
09-Oct-2019	67.4	77.8	0.0	83.3	55.6	27.8	16.7
16-Oct-2019	67.8	83.3	33.3	41.7	75.0	16.7	8.3
23-Oct-2019	67.7	69.2	0.0	38.5	76.9	15.4	7.7
30-Oct-2019	69.6	68.4	15.8	57.9	47.4	36.8	15.8
06-Nov-2019	66.8	84.2	5.3	73.7	84.2	5.3	10.5
13-Nov-2019	67.5	82.4	29.4	52.9	52.9	35.3	11.8
20-Nov-2019	71.7	75.0	6.3	62.5	68.8	12.5	18.8
27-Nov-2019	73.2	42.9	14.3	42.9	71.4	28.6	0.0
04-Dec-2019	64.2	86.4	13.6	59.1	68.2	9.1	22.7
11-Dec-2019	71.3	85.7	28.6	64.3	71.4	21.4	7.1
18-Dec-2019	66.8	70.0	5.0	60.0	70.0	20.0	10.0
08-Jan-2020	67.9	85.0	20.0	75.0	60.0	30.0	10.0
15-Jan-2020	64.9	75.0	6.3	50.0	62.5	25.0	12.5
22-Jan-2020	68.2	50.0	20.0	40.0	45.0	30.0	25.0
29-Jan-2020	67.5	75.0	5.0	55.0	70.0	20.0	10.0

*: Tumour Group **Note:** Although the percentage of low, moderate and high complexity patients are all presented here, the ARIMA model (results presented later in this section) only forecasted the percentage of high complexity patients.

**Table 3:** Forecasted and actual percentage of high symptom complexity level.

Clinic Visit Date	Forecasts	Actual Observations	Absolute Error
05-Feb-2020	14.6%	19.0%	4.4%
12-Feb-2020	20.9%	25.0%	4.1%
19-Feb-2020	7.3%	13.3%	6.0%
26-Feb-2020	24.3%	12.5%	11.8%
04-Mar-2020	14.5%	23.5%	9.0%
11-Mar-2020	14.2%	9.1%	5.1%
18-Mar-2020	20.0%	25.0%	5.0%
25-Mar-2020	26.7%	28.6%	1.9%

**Note:** Mean Absolute Predicting Error = 5.9%.

## Data Availability

The dataset used in the current study is not publicly available due to confidential patient information, but is available from the corresponding author on reasonable request.
